# 

**Published:** 1887-04

**Authors:** 


### CONTENTS, APRIL, 1887, (VOL. II, NO. X.)

Original Articles—Case of Gunshot-Wound of Intestines Resection of Bowels—Death. By Geo. Cupples, M. D. San Antonio, Texas. .......... 401
A Unique Case: an immense Fibroid, involving Uterus and Ovaries, with extensive adhesions; exploratory laparotomy. By A. W. Acheson, M. D., Denison, Tex. 402
Tait's Operation—Recovery. By B. F. Kingsly, M. D. San Antonio. Texas. .......... 404
Two Cases of Curious Sequellas in Rheumatism. By T. C. Osborn, M. D., Cleburne, Texas .......... 405
A Compound Comminuted Fracture of the Frontal Bone, with loss of Brain Substance—Recovery. By R. C. Nettles, M. D., Marlin, Texas .......... 412
A Doctor's Rough Experience with the "Law." By R. G. Williams, M. D., Whitney, Texas. .......... 414
Society Notes—Programme: Texas State Medical Ass'n. 417
Van Zandt County Medical Association. .......... 418
Travis County Medical Society .......... 418
Personal. .......... 418
Editorial—Shall the Majority Rule? .......... 419
No Banquet. .......... 422
What are the Duties of a Publishing Committee? .......... 423
The work of Texas Surgeons. .......... 425
Obituary—Dr. Wm. Jefferson Burt, Late Sec., T. S. M. L. 426
Medical News and Miscellany—Surgery in Texas, as exemplified by the Committee's report .......... 429
Capital Punishment by Electricity .......... 431
Valuable Papers .......... 432
Honors to Prof. Dudley S. Reynolds .......... 432
The Very Latest .......... 433
Exhibits at the Texas Medical Convention .......... 433
The Texas State Pharmaceutical Association .......... 434
Central Texas Medical Association .......... 434
Papers for the Sections .......... 434
What is a Banquet? .......... 435
To the Members Texas State Medical Association .......... 435
To whom it may concern .......... 435
A deserved Compliment .......... 435
Bibliography— .......... 438
Advertisers Notices, to numerous to mention but all interesting and worth reading .......... 440

